# FMRP: a triple threat to PSD-95

**DOI:** 10.3389/fncel.2013.00057

**Published:** 2013-04-30

**Authors:** Cara J. Westmark

**Affiliations:** Department of Neurology, University of WisconsinMadison, WI, USA

Autism is a spectrum of developmental disorders characterized by deficits in verbal and non-verbal communication, social awareness and interactions, and imaginative play (Caronna et al., 2008). There is a strong genetic basis for autism, which is highly comorbid with single-gene disorders including fragile X syndrome (FXS) (Wang et al., 2010). The main challenges that have plagued the field include accurate and early diagnosis, identifying susceptibility genes, defining the cellular and molecular mechanisms through which genetic mutations confer disease risk and phenotypes, and improving interventions and treatments. In their *Cell* article, Tsai and colleagues provide a mechanistic framework explaining how multiple autism-related genes cooperate in experience-dependent synapse elimination and how that mechanism fails in FXS (Tsai et al., 2012). Their results strongly support the contention that bypassing proteasome-mediated degradation of postsynaptic density protein 95 (PSD-95) contributes to altered synaptic plasticity in autism.

Synaptic plasticity is the biological basis for learning and memory and occurs at the postsynaptic density (PSD), a protein dense region at the postsynaptic membrane of excitatory synapses (Sheng and Hoogenraad, 2007). The PSD concentrates and organizes hundreds of proteins including membrane receptors, signaling molecules, and scaffolding proteins. In response to synaptic activity, this dynamic region undergoes structural changes that result in the formation or elimination of dendritic spines. Synapse formation and pruning are critical for synaptic plasticity; yet, the molecular mechanisms that regulate these processes have remained elusive. Tsai and colleagues elegantly demonstrate roles for several autism-related molecules including myocyte enhancer factor 2 (MEF2), protocadherin 10 (Pcdh10), and fragile X mental retardation protein (FMRP) in a proteasome-mediated pathway that degrades PSD-95 and leads to synapse elimination (Tsai et al., 2012).

PSD-95 is a major PSD scaffolding protein with established roles in modulating N-methyl-D-aspartate receptor (NMDAR) signaling, α-amino-3-hydroxy-5-methyl-4-isoxazolepropionic acid receptor (AMPAR) endocytosis, dendritic spine stabilization, and synaptic strength (Keith and El-Husseini, 2008; Woods et al., 2012). PSD-95 abundance is a culmination of protein synthesis, transport, and degradation processes all occurring locally at synapses. PSD-95 synthesis at synapses is regulated through group 1 metabotropic glutamate receptors (mGluR), FMRP, and microRNA-125a (miR-125a) (Todd et al., 2003; Muddashetty et al., 2012) while degradation occurs via proteasomes after protein ubiquitination by the E3 ligase murine double minute 2 (Mdm2) (Colledge et al., 2003). Tsai and colleagues demonstrate that activation of MEF2 results in a significant decrease in PSD-95, which is due to protein degradation and not reduced protein synthesis.

The MEF2 family of transcription factors is highly expressed in brain and key regulators of activity-dependent synapse elimination and learning-induced structural plasticity (Flavell et al., 2006; Cole et al., 2012). MEF2C is the major isoform involved in hippocampal synaptic function (Akhtar et al., 2012), and mutations in the gene occur in 1% of patients with moderate to severe intellectual disability and 2% of patients with Rett syndrome (Zweier and Rauch, 2011). The MEF2 proteins bind to synaptic activity-responsive elements (SARE), which are significantly enriched in genes that encode mRNAs targeted by FMRP (Rodríguez-Tornos et al., 2013). Tsai and colleagues identified the autism-related gene *Pcdh10* (Redies et al., 2012) in both a genome-wide screen of MEF2 transcriptional targets (Flavell et al., 2008) and as an FMRP mRNA target (Darnell et al., 2011). They then confirmed that MEF2 activates transcription of *Pcdh10* and that FMRP associates with *Pcdh10* mRNA. *Pcdh10* encodes a cadherin superfamily protein whose levels change in response to neuronal activity (Morrow et al., 2008), but little was known regarding its function. The authors demonstrate that Pcdh10 is present in spines and at the excitatory synapses and functions to associate ubiquitinated PSD-95 with the proteasome. The degradation of PSD-95 by proteasomes leads to synapse elimination.

This MEF2-, Pcdh10-, Mdm2-dependent pathway for ubiquitin-dependent, proteasome-mediated degradation of PSD-95 goes awry in the absence of FMRP. FMRP is an mRNA binding protein absent or mutated in FXS, a disorder characterized by excessive immature dendritic spines suggesting a deficit in excitatory synapse elimination (Comery et al., 1997). In response to neuronal activity, MEF2 is activated and induces rapid and robust synapse elimination in wild type, but fails to eliminate synapses in *Fmr1*^*KO*^ hippocampal neurons (Pfeiffer et al., 2011). Tsai and colleagues show robust MEF2-activated transcription of *Pchd10* and elevated basal translation of Pchd10 in *Fmr1*^*KO*^ indicating that FMRP functions downstream of MEF2. They further demonstrate that *Fmr1*^*KO*^ neurons exhibit deficits in ubiquitination and degradation of PSD-95 due to decreased colocalization of and interaction between Mdm2 and PSD-95. There is increased interaction between elongation factor 1 alpha (EF1α) and Mdm2 in *Fmr1*^*KO*^ mice and when EF1α is bound to Mdm2 the latter loses its ability to bind to PSD-95. EF1α is an FMRP target mRNA and its protein levels are upregulated in *Fmr1*^*KO*^ brain. Thus, in the absence of FMRP, EF1α is overexpressed and sequesters Mdm2 resulting in decreased ubiquitination and degradation of PSD-95. The authors conclude their study by demonstrating EF1α knockdown in *Fmr1*^*KO*^ neurons restores MEF2-induced synaptic localization of Mdm2, interaction between Mdm2 and PSD-95, PSD-95 degradation and robust synapse elimination. Interestingly, PSD-95 is associated with Williams and Angelman syndromes further establishing a link with multiple neurodevelopmental disorders (Feyder et al., 2010; Cao et al., 2013).

**Figure 1:**
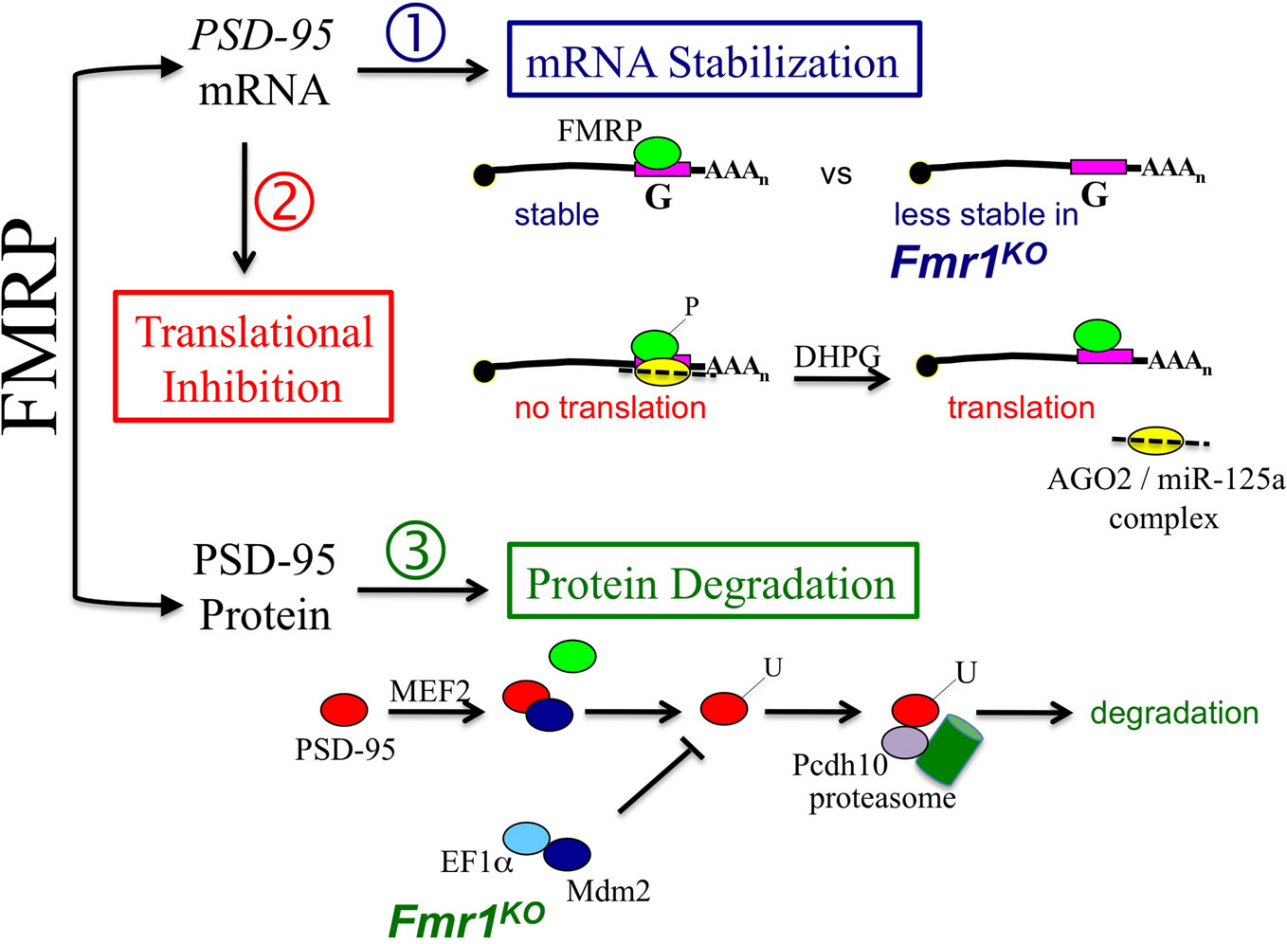
**Fragile X mental retardation protein (FMRP) poses a triple threat to postsynaptic density protein 95 (PSD-95) levels at the synapse.** (1) FMRP binds directly to a guanine (G)-rich sequence within the 3'-untranslated region (UTR) of *PSD-95* mRNA to regulate mRNA stability (Zalfa et al., 2010). (2) Phosphorylated FMRP recruits Argonaute 2 (AGO2) and microRNA-125a (miR-125a) into an inhibitory complex on *PSD-95* mRNA that blocks translation (Muddashetty et al., 2012). Group 1 mGluR stimulation with (*S*)-3,5-dihydroxyphenylglycine (DHPG) leads to dephosphorylation of FMRP, release of AGO2 from the mRNA and activation of translation. And (3) myocyte enhancer factor 2 (MEF2) induces ubiquitination of PSD-95 by the E3 ligase murine double minute 2 (Mdm2), which allows for the association of PSD-95 with protocadherin 10 (Pcdh10) and proteasomes. The degradation of PSD-95 by proteasomes leads to synaptic pruning (Tsai et al., 2012). However, in the absence of FMRP, elongation factor 1 alpha (EF1α) is overexpressed and sequesters Mdm2 resulting in decreased ubiquitination (U) and degradation of PSD-95. It remains to be determined how the multiple functions of FMRP are coordinated in a cell- and/or brain region-specific manner to control synpatic levels of PSD-95.

The Centers for Disease Control estimate autism rates at 1 in 50 school-age children (Blumberg and Bramlett, 2013). It is imperative to understand the underlying cellular and molecular mechanisms in order to design better therapeutics for this epidemic. Tsai and colleagues uncover an integrated process of transcriptional, translational, and protein turnover events through which the activity-dependent transcription factor MEF2 refines synaptic connections in wild type neurons and how that process fails in *Fmr1*^*KO*^. During their studies, the authors define a novel, albeit indirect, role for FMRP in modulating PSD-95 degradation. The convergence of multiple autism-related genes at the level of PSD-95 degradation defines a pivotal pathway that controls synaptic pruning. It remains to be determined how additional functions of FMRP in regards to PSD-95, i.e., mRNA stabilization and translational inhibition, affect synapse remodeling (Figure 1).
